# Supplementary material for: Efficacy and effectiveness of SARS-CoV-2 vaccines for death prevention: A protocol for a systematic review and meta-analysis
Source: PLoS One. 2022 Jul 28;17(7):e0265414. doi: 10.1371/journal.pone.0265414 (PMC9333202; doi:10.1371/journal.pone.0265414)
Supplement: S1 Appendix — (DOCX) [file pone.0265414.s002.docx]

**Appendix I**

List of currently available vaccines as of December 4th, 2021, which have either been approved, authorized, licensed, granted emergency use authorization, or they have been made available for use outside of clinical trials by a regulatory agency, a national authority, or some other entity (1):

1. Anhui Zhifei Longcom, ZF2001, protein subunit
2. Bharat Biotech, Covaxin, inactivated
3. CanSino, Ad5-nCoV, non-replicating viral vector
4. Center for Genetic Engineering and Biotechnology (CIGB), CIGB-66, protein subunit
5. Chumakov Center, KoviVac, inactivated
6. FBRI, EpiVacCorona, protein subunit
7. Gamaleya, Sputnik Light, non-replicating viral vector
8. Gamaleya, Sputnik V, non-replicating viral vector
9. Instituto Finlay de Vacunas Cuba, Soberana 02, protein subunit
10. Instituto Finlay de Vacunas Cuba, Soberana Plus, protein subunit
11. Janssen (Johnson & Johnson), Ad26.COV2.S, non-replicating viral vector
12. Kazakhstan RIBSP, QazVac, inactivated
13. Medigen, MVC-COV1901, protein subunit
14. Minhai Biotechnology Co, SARS-CoV-2 Vaccine (Vero Cells), inactivated
15. Moderna, mRNA-1273, RNA
16. Oxford/AstraZeneca, AZD1222, non-replicating viral vector
17. Pfizer/BioNTech, BNT162b2, RNA
18. Serum Institute of India, Covishield (Oxford/Astrazeneca formulation), non-replicating viral vector
19. Serum Institute of India, COVOVAX (Novavax formulation), protein subunit
20. Shifa Pharmed Industrial Co, COVID-19 Inactivated Vaccine, inactivated
21. Sinopharm (Beijing), BBIBP-CorV (Vero Cells), inactivated
22. Sinopharm (Wuhan), Inactivated (Vero Cells), inactivated
23. Sinovac, CoronaVac, inactivated
24. Takeda, TAK-919 (Moderna formulation), RNA
25. Vaxine/Cinnagen Co., COVAX-19, protein subunit
26. Zydus Cadila, ZyCoV-D, DNA

**Appendix II**

Preliminary search strategy with Ovid MEDLINE

Total: 2861

1. exp COVID-19/
2. Severe Acute Respiratory Syndrome/ or exp SARS-CoV-2/ or SARS Virus/
3. Coronavirus Infections/
4. covid.mp.
5. sars-cov-2.mp.
6. 1 or 2 or 3 or 4 or 5
7. vaccines/ or viral vaccines/ or exp covid-19 vaccines/
8. immunotherapy/ or immunization/ or vaccination/
9. immunology.mp.
10. Drug Development/
11. (Moderna or mRNA-1273).mp.
12. pfizer/ or pfizer.mp.
13. (BioNTech or BNT162b2 or Tozinameran or Comirnaty).mp.
14. (Oxford AstraZeneca or Covishield or AZD1222).mp.
15. Ad5-nCoV.mp.
16. BBIBP-CorV.mp.
17. (CoronaVac or PiCoVacc).mp.
18. Sputnik V.mp.
19. Johnson & Johnson.mp.
20. RBD-Dimer.mp.
21. Covaxin.mp.
22. 7 or 8 or 9 or 10 or 11 or 12 or 13 or 14 or 15 or 16 or 17 or 18 or 19 or 20 or 21
23. Patient Safety/ or Safety/ or safety.mp.
24. treatment outcome/ or disease-free survival/ or sustained virologic response/
25. adverse event.mp.
26. "Drug-Related Side Effects and Adverse Reactions"/
27. (efficacy or effectiveness).mp.
28. outcome.mp.
29. death.mp. or Death/
30. mortality/ or "cause of death"/ or fatal outcome/ or survival rate/
31. all-cause mortality.mp.
32. 23 or 24 or 25 or 26 or 27 or 28 or 29 or 30 or 31
33. 6 and 22 and 32

Image of search strategy from Ovid MEDLINE:


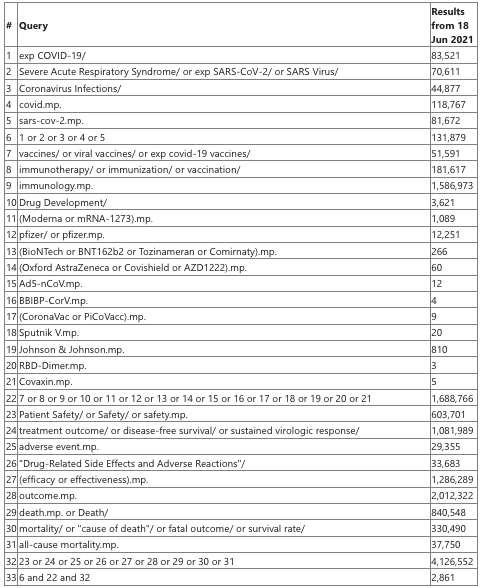


**Appendix III**

Preliminary data extraction list

1. General study information: title, authors, year of publication or publication status, type of publication, country/countries and country level income, conflicts of interest, sources of funding. If information on background including variants of concern in the study period are not available in the paper, we will try to get this information in publicly available websites.

2. Study characteristics: design, setting, follow-up period, recruitment procedures (randomization, concealment, blinding)

3. Patient characteristics: number of participants in intervention or control group, sex, age, exposure (background) risk, comorbidities, previous COVID infection

4. Intervention and COVID-related data: vaccine name and technology, developer, number of doses and/or boosters, date of intervention, variant of virus, wave of epidemic, viral gene sequencing in country, case definition

5. Outcome/results (for each subgroup where appropriate):

a. Primary outcome: whether reported, definition in study

b. Secondary outcome: whether reported

c. Statistical technique used, types of analysis done, results of analysis (odds ratio, risk ratio, confidence intervals, p-values)

6. Miscellaneous

References

1. COVID 19 vaccine tracker [Internet]. [cited December 4 2021] Available from: <https://covid19.trackvaccines.org/vaccines/approved/>
