# Supplementary material for: Efficacy and effectiveness of SARS-CoV-2 vaccines for death prevention: A protocol for a systematic review and meta-analysis
Source: PLoS One. 2022 Jul 28;17(7):e0265414. doi: 10.1371/journal.pone.0265414 (PMC9333202; doi:10.1371/journal.pone.0265414)
Supplement: Attachment — Submitted filename: Review_Comments.docx [file pone.0265414.s003.docx]

The authors have written the protocol to study a very critical estimate with respect to COVID-19 vaccines. I thank the authors for this study as its essential in coming months and years to come. I am really excited and look forward to the results from this review. Wish them all the best. I have few suggestions and comments:

1) The authors can also add the WHO Global research for COVID-19 database in the search strategy. It’s exhaustive and covers all languages across the world.

2) For reference 6, I would also recommend looking into the LHSTM vaccine landscape as it started well before WHO initiated its dashboard. This reference would also be useful to pool results of different vaccine trials across different studies.

3) Apart from citing reference 18, I would also consider non availability of timely genomic sequencing data across several countries to be a confounder as vaccines tend to show different efficacy/real world effectiveness across strains. I would be interested to see whether the authors would consider a sub-group analysis on this aspect. (Agree that this data is not well published across all countries and can be a potential limitation in this study as well as core recommendation for global public health policy).

4) Reference 22 is a potential cross validation to the results you will be getting in your research.

5) By mentioning cohort studies, I believe vaccine challenge studies would also be included.

6) By mentioning case-control studies, I believe test negative studies would be included.

7) The Disease definition needs to be looked into very carefully as it determines the end point of the review. Several countries tend to use different cut off points for cT values. It would be interesting to see this in sub-group analysis (if the cut-off is mentioned in the study).

8) After reading the objectives, I get a sense that only research articles would be reviewed. There are countries like India that show deaths between vaccinated and non-vaccinated in a public website. Will such sources be included?. I stress this because as the authors mentioned, most studies see primary outcomes like hospitalization, Oxygen use, ICU admission, etc.

9) I think for all-cause mortality a cut-off for 4 weeks would be ideal as this is how post-illness sequelae deaths are classified in Influenza and other respiratory viruses. This cut –off was played around by several countries to get their reported death numbers down.

10) I noticed you had mentioned that all-cause mortality was taken primarily due to the poor COVID-19 attribution/certification in several countries. How do you plan to address the poor classification of death as an adverse event in several countries?

11) Suggest using Zotero instead of EndNote. Chances of duplication are there in EndNote. This was my experience. I leave the choice to the authors.

12) I would like to have more details on the electronic data extraction form. What are the added benefits of using this as compared to traditional methods ?

13) Point 3 is addressed in your methods. Thank you

14) I am surprised why low quality studies are excluded and then included in sensitivity analysis. I get the authors thought behind this. You can rephrase the statement as “sensitivity analysis would be done among the included studies in scenario 1. In scenario 2 we would explore how low quality studies would have affected the results.
